# Uncommon presentation of allergic bronchopulmonary aspergillosis during the COVID-19 lockdown: a case report

**DOI:** 10.1186/s12890-020-01373-7

**Published:** 2020-12-29

**Authors:** Daniela Savi, Giada Valente, Alessandra Iacovelli, Federica Olmati, Mario Bezzi, Paolo Palange

**Affiliations:** 1 Department of Public Health and Infectious Diseases, “Sapienza” University of Rome, 00185 Rome, Italy; 2 Department of Radiology, Oncology and Pathology, “Sapienza” University of Rome, 00185 Rome, Italy

**Keywords:** Coronavirus disease 2019 (COVID-19), Allergic bronchopulmonary aspergillosis (ABPA), *Aspergillus*-associated respiratory disorders

## Abstract

**Background:**

During the ongoing pandemic of coronavirus disease 2019 (COVID-19), lockdown periods have changed the way that people and communities live, work and interact.

**Case presentation:**

This case report describes an uncommon but important presentation of allergic bronchopulmonary aspergillosis (ABPA) in a previously healthy male, who decided to live in the basement of his house when Italy entered a nationwide lockdown during the COVID-19 pandemic. As high resolution computed tomography (HRCT) of the chest on admission showed diffuse miliary nodules, a miliary tuberculosis was initially suspected. However, further investigations provided a diagnosis of unusual presentation of ABPA.

**Conclusions:**

This case highlights the importance of maintaining awareness of *Aspergillus*-associated respiratory disorders during the COVID-19 pandemic, especially because lifestyle changes associated with home isolation carry an increased risk of exposure to mold spores present in some indoor environments.

## Background

It has been recognized that, in addition to its detrimental effects on the lungs and other organs, coronavirus disease 2019 (COVID-19) can have direct and indirect psychological and social consequences that impact mental health [1]. For example, the associated periods of quarantine can result in depressive symptoms and sleep disturbance, leading to behavioral changes in the population [1]. This case report describes an uncommon but important presentation of allergic bronchopulmonary aspergillosis (ABPA) in a male, who decided to live in the basement of his house when Italy entered a nationwide lockdown during the COVID-19 pandemic.

## Case presentation

**Fig. 1:**
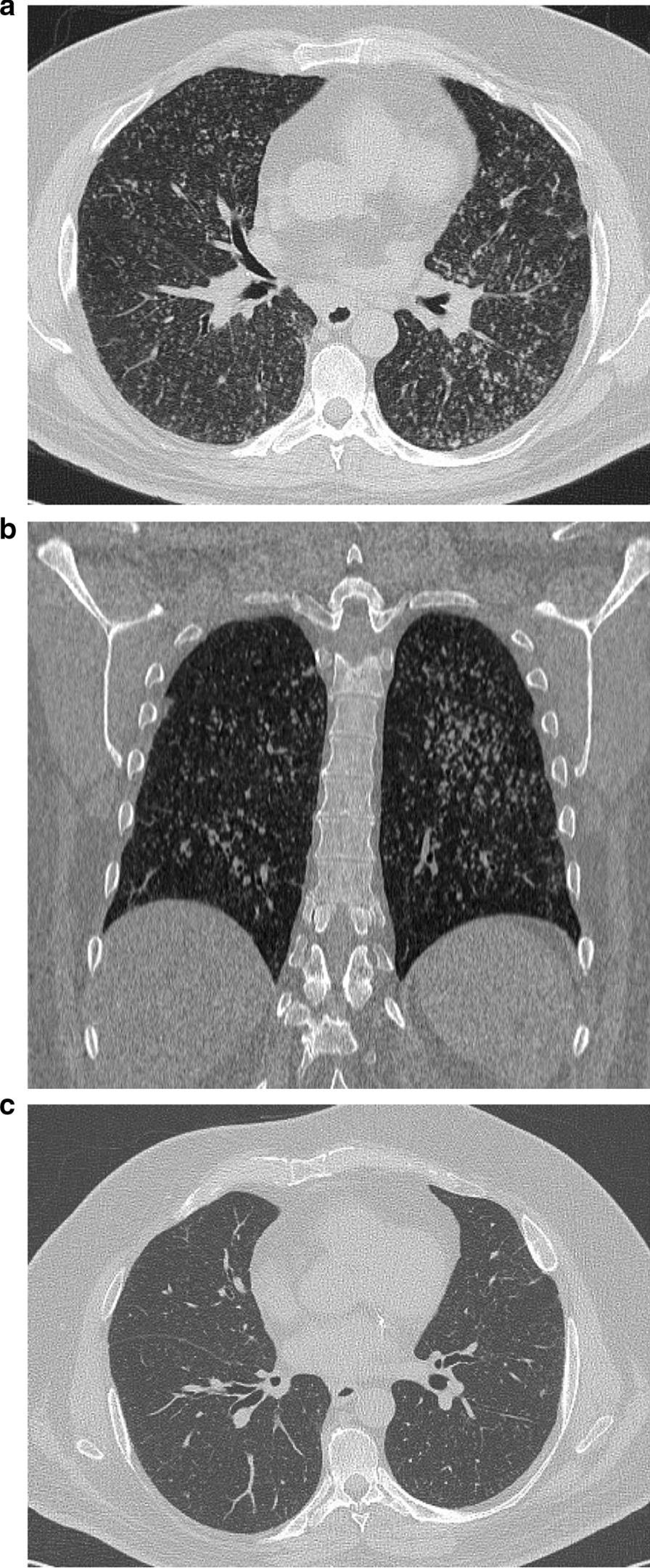
High resolution computed tomography. **a** Computed tomography axial scan of chest showing multiple small hazy nodular opacities diffusely distributed throughout both lung fields; **b** computed tomography coronal scan of chest showing an overlapping "tree- in- bud" appearance in the lower field of the left lung; **c** computed tomography axial scan of chest three months after hospital admission showing reduction of the multiple nodular opacities

During the COVID-19 pandemic, in April 2020, a 55-year-old Caucasian man presented to the emergency department of our institution with fever up to 38.6 °C, cough and shortness of breath. He had a history of ischemic heart disease, diabetes, arterial hypertension, severe obesity, asthma, and he was a smoker. Because of respiratory failure, oxygen therapy was promptly administered via a Venturi mask at 35%. Bilateral crackles were present during chest auscultation; there were no other relevant findings on physical examination. Blood chemistry (Table 1) revealed a high white cell count and high levels of both C-reactive protein (CRP) and D-dimer (the latter being 1652 mcg/l; normal: 0–550 mcg/l). Due to the ongoing pandemic and based on his symptoms, the patient was initially managed as a suspected case of COVID-19, even though two consecutive nasopharyngeal swabs were negative for SARS-CoV-2. High resolution computed tomography (HRCT) of the chest, before and after intravenous injection of iodinated contrast medium, was obtained. The exam showed multiple small hazy nodular opacities diffusely distributed throughout both lung fields, with no specific lobar preference (Fig. 1a, b). The nodules were centrilobular, with a linear branching pattern and a “tree-in-bud” appearance, mainly visible in the lower left lung field (Fig. 1b). CT also showed slightly enlarged mediastinal lymph nodes, without sign of necrosis, of a suspected reactive/inflammatory nature. The HRCT findings were not considered indicative of COVID-19 pneumonia.

**Table 1:** Blood results during admission

Day of admission	White cell count (× 10^9^/L)	Neutrophils (× 10^9^/L)	Lymphocytes (× 10^9^/L)	Eosinophils (× 10^9^/L)	CRP (mg/dl)	D-dimer (mcg/l)	Total IgE (IU ml^−1^)	IgE *A. fumigatus* *(KU/L)*	IgG *A. fumigatus* (mg/l)
Day 1	12.08	10.47	0.72	0.20	31,15	1652			
Day 21	5.97	4.48	0.94	0.40	0.9	1257	1016	0.09	52,7

*CRP* C- reactive protein, *IgE* serum immunoglobulin E, *IgG* serum immunoglobulin G

A possible diagnosis of miliary tuberculosis or other diffuse infectious/inflammatory disease was made and the patient was transferred to our respiratory unit for further investigations. Past medical history revealed no hemoptysis, chest pain or weight loss. The patient reported that during the pandemic he had decided to live in the damp basement of his house, to be isolated from his family, and that he used to work there for several hours a day. This occurred six weeks before the onset of symptoms and it has been the only major change in his daily life.

Sputum was negative for acid-fast bacilli in three specimens, and both Mantoux and QuantiFERON tests were negative, such that a diagnosis of tuberculosis was excluded. Pneumonia due to Legionella *sp*., Mycoplasma *sp.* or Chlamydia *sp.* infection was excluded as were HIV infection and autoimmune disease. Another possible diagnosis was secondary neoplasm of the lung, but a whole-body CT scan did not reveal any malignancy. An antibiotic therapy course was empirically started with ceftriaxone 2 g a day and azithromycin 500 mg once a day for seven days. Three weeks after the hospital admission, the patient repeated a non-enhanced chest CT scan that showed persistence of the pattern described above. Antinuclear and antineutrophilic cytoplasmic antibody tests were negative. The patient’s clinical condition slightly improved: he did not present with fever, and oxygen therapy via Venturi mask was promptly reduced to 24%. Blood chemistry showed a normal white cell count and reduced CRP (Table 1). The patient underwent bronchoscopy and the bronchial lavage fluid (BLF) revealed a galactomannan level of 3.7 (upper normal limit: 0.5). Total serum immunoglobulin E (IgE) levels were raised (1016 IU ml^−1^; normal: < 100 IU ml^−1^), although IgE specific for *A. fumigatus* was negative, while IgG specific for *A. fumigatus* was also high (Table 1). Spirometry showed an obstructive ventilatory pattern, reversible after salbutamol administration, confirming a diagnosis of asthma. Forced vital capacity (FVC) was 3.22 l (70% of predicted) and a forced expiratory volume in 1 s (FEV1) was 2.23 l (61% of predicted) and 3.00 l (+ 34.4%) after bronchodilatation. BLF culture was positive for *Pseudomonas aeruginosa rugosa* and *Achromobacter xylosoxidans*. Finally, the following criteria for diagnosis of ABPA were satisfied: (1) predisposing condition as bronchial asthma; (2) elevated total IgE levels (> 1000 IU ml^−1^); (3) serum IgG antibodies against *A. fumigatus*; (4) radiographic pulmonary opacities consistent with ABPA. Immediate cutaneous hypersensitivity to *Aspergillus* antigen was not performed because the patient was on systemic antihistamines. The patient was started on prednisolone (0.5 mg/kg/day) for four weeks. To reduce the antigen burden, oral isavuconazole therapy was also started with a loading dose of 200 mg tid for the first three days and then 200 mg once a day for eight weeks. He was discharged two months after the hospital admission with improved clinical conditions, without oxygen therapy and with reduced total serum IgE levels of 306 IU ml^−1^. The patient continued steroids, which were gradually tapered down every two weeks. Three months after hospital admission, the patient was seen in our outpatient clinic and he referred only to mild dyspnea during physical exercise. HRCT of the chest showed significant reduction of the multiple nodular opacities (Fig. 1c) and a lung pattern that had almost returned to normal appearance. Total IgE levels were 335 IUml^−1^. Prednisolone was also tapered, and it is still ongoing at the time of this case presentation at 10 mg daily.

## Discussion and conclusions

ABPA is a hypersensitivity disease of the lung resulting from an immune response (IgE mediated) to antigens of the *Aspergillus* species, in particular *A. fumigatus*. The pathogenesis of ABPA is complex and can involve both genetic and immune factors. It occurs in adult patients with asthma and in all ages of patients with cystic fibrosis [2]. The main cause has been associated with exposure to high concentrations of spores, in damp and wet buildings and even outdoors in certain places, manifesting with poorly controlled asthma, recurrent pulmonary infiltrates and bronchiectasis [2]. The disease remains under-diagnosed in many countries, and as many as one-third of cases are misdiagnosed as pulmonary tuberculosis [3]. HRCT findings in ABPA consist of bronchiectasis, mucoid impaction, and centrilobular nodules with a tree-in-bud pattern. Pleural involvement is less common, manifesting with effusion and pleural thickening. Fibrotic changes and even end-stage fibrosis may develop [3]. It is important after diagnosis of ABPA to prevent or delay the development of bronchiectasis, one manifestation of permanent lung damage in ABPA.

The diagnosis relies on combining the clinical picture with evidence of a hypersensitivity reaction to *Aspergillus*. As no single criterion is discriminatory, guidelines have been developed to help define the combination of clinical, radiological, and immunological features that lead to a diagnosis of ABPA [4].

There are five stages of ABPA (acute, remission, exacerbation, corticosteroid-dependent asthma, fibrotic lung disease), but in none of these stages is the miliary pattern currently described [5]. Radiographic images of ABPA vary depending on the stage of the disease. For example, during the acute stage there may be homogenous infiltrates, mucus plugging, lobar consolidation, “tree-in-bud” appearance, and bronchiectasis [6]. HRCT usually reveals central bronchiectasis (CB) in ABPA patients.

This case is interesting both from a radiological and a sociological point of view: the misleading ABPA presentation and the role of home isolation due to COVID-19 lockdown. From a review of the literature, this is the fourth case of ABPA presenting as randomly scattered and hazy nodules distributed throughout both lung fields [7–9]. The unusual radiological findings, i.e. a miliary pattern and centrilobular nodules, has often been responsible for misdiagnosis of ABPA as pulmonary tuberculosis (PTB).

Moreover, this case demonstrates the social effects of isolation due to lockdown during the COVID-19 pandemic. Among the consequences of quarantine, our patient changed his daily life with new behavior characterized, for example, by smart working and an increased need for privacy. For susceptible hosts, such as our patient, spending a prolonged time in damp rooms could lead to repeated inhalation of *Aspergillus* spores and consequent airways colonization eliciting an allergic response. In this case, a particular environment that resulted in exposure to *A. fumigatus* was crucial.

The ongoing COVID-19 pandemic is a strong reminder that lockdown periods changed the way people and communities live, work, and interact, and underlines the need to ensure the living and working environment comfortable, for both outdoor and indoor spaces. A recent study highlighted the relevance of maximum flexibility of living spaces (ground floors, basements, free floors), so that they can easily be adapted to provide, for example, a temporary quiet work station, and to maintain social distancing [10].

In conclusion, the diagnosis of ABPA can be difficult as not all criteria are always satisfied, and the disease can have various radiological manifestations. A review of the literature shows that presentation with a computed tomography showing only randomly scattered nodules with some degree of tree-in-bud appearance is not typical of ABPA, suggesting that this pattern should be added to descriptions of the acute stage of the disease. Early diagnosis and management of ABPA in such cases will help to prevent the development of end-stage pulmonary fibrosis. This case highlights the importance of a high level of awareness of the possibility of *Aspergillus*-associated respiratory disorders during a COVID-19 pandemic, especially because lifestyle changes associated with home isolation can increase the risk of exposure to mold spores, which are present in some indoor environments.

## Data Availability

All data generated or analysed during this study are included in this published article.

## Abbreviations

COVID-19: Coronavirus disease 2019
ABPA: Allergic bronchopulmonary aspergillosis
HRCT: High resolution computed tomography
FVC: Forced vital capacity
FEV1: Forced expiratory volume in 1 s
